# Interleukin-6 increases inner cell mass numbers in bovine embryos

**DOI:** 10.1186/s12861-019-0182-z

**Published:** 2019-02-01

**Authors:** Lydia K. Wooldridge, Alan D. Ealy

**Affiliations:** Department of Animal and Poultry Sciences, Virginia Polytechnic Institute and State University, 3430 Litton-Reaves Hall (0306), Blacksburg, VA 24060 USA

**Keywords:** Embryo, In vitro embryo production, Blastocyst, Cytokine, Inner cell mass

## Abstract

**Background:**

Work in other species suggests that interleukin-6 (IL6) promotes early embryo development. It was unclear whether IL6 serves as an embryokine in cultured bovine embryos. This work was undertaken to elucidate the role of IL6 during in vitro bovine embryo production.

**Results:**

Transcripts for *IL6* and its two cognate receptor subunits (*IL6R*, *IL6ST*) were confirmed in bovine embryos from the 1-cell to blastocyst stages. Supplementing 100 ng/ml recombinant bovine IL6 to in vitro-produced bovine embryos at day 1, 3 or 5 increased (*P* < 0.05) inner cell mass (ICM) cell number and the ICM:trophectoderm (TE) ratio but not TE cell number. No increase in ICM or TE cell number was observed after supplementation of 1 or 10 ng/ml IL6 beginning at either day 1 or 5. Sequential supplementation with 100 ng/ml IL6 at both day 1 and 5 (for a total of 200 ng/ml IL6) increased (*P* < 0.05) ICM cell number to a greater extent than supplementing IL6 at a single time period in one study but not a second study. Additionally, providing 200 ng/ml IL6 beginning at day 1 or 5 yielded no further increase on ICM cell numbers when compared to supplementing with 100 ng/ml IL6. IL6 treatment had no effect on cleavage or blastocyst formation in group culture. However, IL6 supplementation increased cleavage and day 8 blastocyst formation when bovine embryos were cultured individually.

**Conclusions:**

These results implicate IL6 as an embryokine that specifically increases ICM cell numbers in bovine embryos and facilitates bovine blastocyst development in embryos cultured individually.

## Background

The first lineage specification event during mammalian embryogenesis is the differentiation of the trophectoderm (TE) and the inner cell mass (ICM). The TE will from the outermost layer of the fetal portion of the placenta and the ICM develops into hypoblast and epiblast lineages. The hypoblast will contribute to the extraembryonic endoderm, which forms the yolk sac, while the epiblast gives rise to the three embryonic germ layers and additional extraembryonic lineages. Proper ICM specification and development is crucial to embryo survival. Loss of either ICM lineage is embryonic lethal in mice [1, 2].

In humans, a prominent ICM, as assessed microscopically, is associated with reduced early embryonic loss and increased implantation and live birth rates [3–5]. A similar scenario also exists in cattle, where the current consensus is that bovine embryo culture conditions fail to adequately promote proper ICM development, and this contributes to at least some of the pregnancy losses that occur after transfer of in vitro-produced (IVP) bovine embryos [6, 7]. Bovine IVP blastocysts have fewer ICM cells, elevated apoptosis in the ICM, and produce smaller embryonic disks than their in vivo-produced counterparts [8, 9]. In some cases, embryonic disks could not be detected in IVP conceptuses [6, 10–12]. Also, fewer pregnancies are maintained by IVP conceptuses that lack visible embryonic disks when compared with IVP conceptuses containing prominent embryonic disks [6]. Unfortunately, the embryonic and uterine-derived factors controlling ICM specification and development remain largely unknown in cattle, humans and other mammals.

Two members of the interleukin-6 (IL6) family of cytokines, IL6 and leukemia inhibitory factor (LIF), have been identified as important mediators of embryonic cell development and maintenance. Outside of embryonic development, these two cytokines are best known for their roles in inflammation, cancer, metabolism, and placental development and implantation [13, 14]. Both IL6 and LIF are also known for their abilities to maintain murine embryonic stem cells, which are derived from the ICM of murine blastocysts, by initiating the signal transducer and activator of transcription 3 (STAT3) signaling cascade [15–17]. Both IL6 and LIF can activate STAT3 signaling in various cell types, however, each ligand utilizes a ligand-specific receptor subunit (IL6R or LIFR) and a common subunit that contains the signal transducing regions (IL6ST, GP130). Specifically, LIF requires a heterodimer of IL6ST and LIFR while IL6 uses a heterotrimer composed of two IL6ST subunits and one IL6R subunit [13, 18].

Bovine IVP embryo responses to LIF supplementation are varied, and the effects of IL6 on IVP bovine embryo production have not been explored [19–22]. An embryotrophic role for IL6 is suggested in other species. In the pig, IL6 supplementation during culture increased parthenogenetic blastocyst formation and ICM cell numbers [23]. In the mouse, IL6, and not LIF, appears to be responsible for blastocyst-stage nuclear STAT3 activity in the ICM [24]. Six studies were completed to test the hypothesis that IL6 supplementation promotes blastocyst formation and ICM development in bovine preimplantation embryos.

## Results

### Transcript profiling

**Fig. 1:**
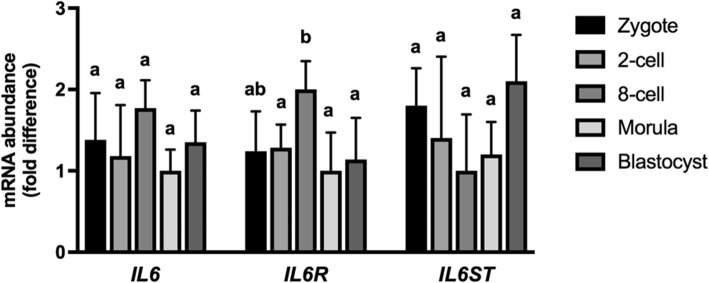
Transcript abundances for *IL6*, *IL6R* and *IL6ST* from zygotes, 2-cell embryos, 8-cell embryos, morulae and blastocysts. Total RNA was isolated from 3 to 5 pools of 10 embryos from each developmental stage before reverse transcription. The relative abundance of each target transcript is expressed as fold change from the embryo stage containing the lowest abundance for the specified transcript by using the 2^[-ddCt]^ approach. Corresponding means and SEMs are indicated by the bars. Different superscripts within each transcript indicates differences (*P* < 0.05)

Transcripts for *IL6* and *IL6R* were detected in each RNA preparation examined at the zygote, 2-cell, 8–16 cell, compact morula and blastocyst stages. Transcripts for *IL6ST* were detected in each of the zygote and blastocyst samples, in 4 of 5 8–16 cell pools, and in 3 of 5 compact morula stage pools. Neither *IL6* nor *IL6ST* transcript abundance were altered across each of the stages (Fig. 1). However, the abundance of *IL6R* transcripts was greater (*P* < 0.05) at the 8-cell stage than at the 2-cell, morula or blastocyst stages (Fig. 1).

### Study a: IL6 treatment at day 5 post-fertilization

In this first study, recombinant bovine IL6 was supplemented from day 5 to 8 post-fertilization. The day 5 time point was chosen because it corresponds with the initiation of blastomere compaction and ICM and TE specification in bovine IVP embryos [25, 26]. A dose-response study was completed using IL6 concentrations based on work completed in porcine embryos [23]. None of these concentrations affected the percentage of embryos which formed blastocysts at day 7 or 8 (Table 1). Exposure to 1 or 10 ng/ml IL6 did not affect ICM, TE, or total cell numbers or the ICM:TE ratio. However, 100 ng/ml IL6 increased both ICM and total cell numbers (*P* < 0.05) (Table 2). The number of TE cells remained unchanged, but the ICM:TE ratio was increased (*P* < 0.05) in this treatment group.

**Table 1:** Cleavage and blastocyst formation across each study

Study	Treatment	Treatment dates(s)	IVF replicates	Total embryos	Cleavage*	Day 7 blastocyst*	Day 8 blastocyst*
A	0 ng/ml IL6	Day 5	4	317	N/A	12.8 ± 1.3^a^	21.8 ± 3^a^
	1 ng/ml IL6	Day 5		316		10.8 ± 1.4^a^	21.8 ± 3.7^a^
	10 ng/ml IL6	Day 5		323		16.8 ± 3.3^a^	23.7 ± 4.6^a^
	100 ng/ml IL6	Day 5		320		12.7 ± 4.2^a^	23.7 ± 6.3^a^
B	0 ng/ml IL6	Day 3	6	240	N/A	10.8 ± 4^a^	24.2 ± 6.3^a^
	100 ng/ml IL6	Day 3		240		14.6 ± 3.4^a^	30.4 ± 6.8^a^
	100 ng/ml IL6	Day 5		240		13.8 ± 3.3^a^	25.8 ± 6.9^a^
	100 + 100 ng/ml IL6	Day 3 + 5		240		12.5 ± 4.8^a^	32.9 ± 6.4^a†^
C	0 ng/ml IL6	Day 1	4	249	73.4 ± 3.7^a^	12.3 ± 4.2^a^	20.1 ± 3.5^a^
	1 ng/ml IL6	Day 1		297	80.1 ± 5.9^a†^	17.9 ± 2.5^a^	27.1 ± 2.4^a^
	10 ng/ml IL6	Day 1		301	76.4 ± 5.6^a^	18.1 ± 3.7^a^	27.7 ± 4.7^a^
	100 ng/ml IL6	Day 1		248	76.4 ± 3.6^a^	19.4 ± 4.1^a^	29.6 ± 4.2^a^
D	0 ng/ml IL6	Day 1	6	395	74.8 ± 4.5^a^	11.5 ± 1 ^a^	20.3 ± 2.1 ^a^
	100 ng/ml IL6	Day 1		437	76.8 ± 2.2^a^	10.8 ± 1.7 ^a^	21.5 ± 2.5 ^a^
	100 ng/ml IL6	Day 5		392	77.8 ± 3.8^a^	10.9 ± 1 ^a^	21.6 ± 2.4 ^a^
	100 + 100 ng/ml IL6	Day 1 + 5		440	75.2 ± 5.2^a^	10.2 ± 1.8 ^a^	19.4 ± 3.7 ^a^
	200 ng/ml IL6	Day 1		418	76.7 ± 3.7^a^	9.8 ± 1.6 ^a^	18.9 ± 1.7 ^a^
E	0 ng/ml IL6	Day 5	4	347	87.7 ± 0.1^a^	15.8 ± 0.1^a^	27.9 ± 0.1^a^
	100 ng/ml IL6	Day 5		337	85.3 ± 0.1^a^	17.4 ± 0.1^a^	26.1 ± 0.1^a^
	200 ng/ml IL6	Day 5		392	83.9 ± 0.1^a^	20.5 ± 0.1^a^	30.4 ± 0.1^a^
	100 + 100 ng/ml IL6	Day 1 + 5		357	81.1 ± 0.1^a^	18.9 ± 0.1^a^	29.1 ± 0.1^a^
F	0 ng/ml IL6 (Group)	Day 1	4	175	138/175^a^	17/138^a^	29/138^a^
	Individual						
	0 ng/ml	Day 1		100	65/100^b^	0/65^b^	0/65^b^
	100 + 100 ng/ml IL6	Day 1 + 4		100	79/100^a^	0/79^b^	7/79^c^
	200 ng/ml IL6	Day 1		100	73/100^ab^	0/73^b^	7/73^c^

Different superscripts within each study denote differences. Significance established at *P* < 0.05

† indicates a trend (P = 0.06 in study B and P = 0.09 in Study C) when compared with the control)

*Data for studies A-E are presented as the Mean% ± SEM. Data from study F are presented as proportions

**Table 2:** Embryonic ICM and TE cell counts in Day 8 blastocysts

Study	Treatment	Treatment date(s)	IVF replicates	Blastocysts examined	ICM number	TE number	Total number	ICM:TE Ratio
A	0 ng/ml IL6	Day 5	4	39	53.9 ± 4.2^a^	113.5 ± 5.1^a^	167.4 ± 8.3^a^	0.47 ± 0.03^a^
	1 ng/ml IL6	Day 5		35	52.2 ± 3.3^a^	108.6 ± 6.3^a^	160.8 ± 8.0^a^	0.51 ± 0.04^a^
	10 ng/ml IL6	Day 5		34	60 ± 3.6^a^	116.2 ± 6.5^a^	176.2 ± 8.2^a^	0.56 ± 0.05^a^
	100 ng/ml IL6	Day 5		34	98 ± 6.5^b^	113.2 ± 6.3^a^	211.2 ± 10.3^b^	0.92 ± 0.07^b^
B	0 ng/ml IL6	Day 3	3	16	36.8 ± 4.3^a^	95.6 ± 8.1^a^	132.5 ± 11.7^a^	0.38 ± 0.03^a^
	100 ng/ml IL6	Day 3		11	61.8 ± 8.1^b^	106.3 ± 10.6^a^	168.1 ± 14.0^a†^	0.65 ± 0.11^b^
	100 ng/ml IL6	Day 5		11	58.9 ± 7.6^b^	97 ± 16.5^a^	155.9 ± 22.5^a^	0.7 ± 0.09^b^
	100 + 100 ng/ml IL6	Day 3 + 5		12	74.3 ± 8.7^b^	87.3 ± 7.2^a^	161.5 ± 13.4^a^	0.87 ± 0.10^c^
C	0 ng/ml IL6	Day 1	4	27	56.4 ± 4.1^a^	111 ± 7.9^a^	167.3 ± 9.8^a^	0.56 ± 0.05^ab^
	1 ng/ml IL6	Day 1		39	52.5 ± 3.0^a^	111.7 ± 5.4^a^	164.2 ± 7.3^a^	0.49 ± 0.03^a^
	10 ng/ml IL6	Day 1		39	59.3 ± 3.9^a^	102.3 ± 4.7^a^	161.6 ± 6.8^a^	0.62 ± 0.05^b^
	100 ng/ml IL6	Day 1		41	88.3 ± 4.6^b^	112.7 ± 5.0^a^	200.9 ± 7.6^b^	0.82 ± 0.05^c^
D	0 ng/ml IL6	Day 1	5	28	46.4 ± 3.6^a^	99.6 ± 7.2^a^	146 ± 8.6^a^	0.52 ± 0.05^a^
	100 ng/ml IL6	Day 1		27	79.2 ± 4.7^b^	112.1 ± 7.0^a^	191.3 ± 10.2^b^	0.75 ± 0.05^b^
	100 ng/ml IL6	Day 5		28	79.4 ± 6.0^b^	108.1 ± 6.6^a^	187.5 ± 10.6^b^	0.77 ± 0.05^b^
	100 + 100 ng/ml IL6	Day 1 + 5		28	100 ± 8.4^c^	106.2 ± 4.8^a^	206.2 ± 10.1^b^	0.98 ± 0.09^c^
	200 ng/ml IL6	Day 1		27	77.4 ± 6.9^b^	104.5 ± 5.9^a^	181.9 ± 11.3^b^	0.75 ± 0.06^b^
E	0 ng/ml IL6	Day 5	4	41	56.4 ± 4.1^a^	112.3 ± 7.5^a^	168 ± 10.3^a^	0.53 ± 0.03^a^
	100 ng/ml IL6	Day 5		38	88.7 ± 6.2^b^	111.4 ± 6.9^a^	200.1 ± 8.4^b^	0.83 ± 0.05^b^
	200 ng/ml IL6	Day 5		44	93.7 ± 5.2^b^	116.6 ± 5^a^	210.3 ± 8.4^b^	0.83 ± 0.05^b^
	100 + 100 ng/ml IL6	Day 1 + 5		44	89.8 ± 5.7^b^	115 ± 6.5^a^	204.8 ± 10.5^b^	0.81 ± 0.05^b^

Different superscripts in each study denote differences. Significance established at *P* < 0.05

† indicates a trend (*P* = 0.09)

**Fig. 2:**
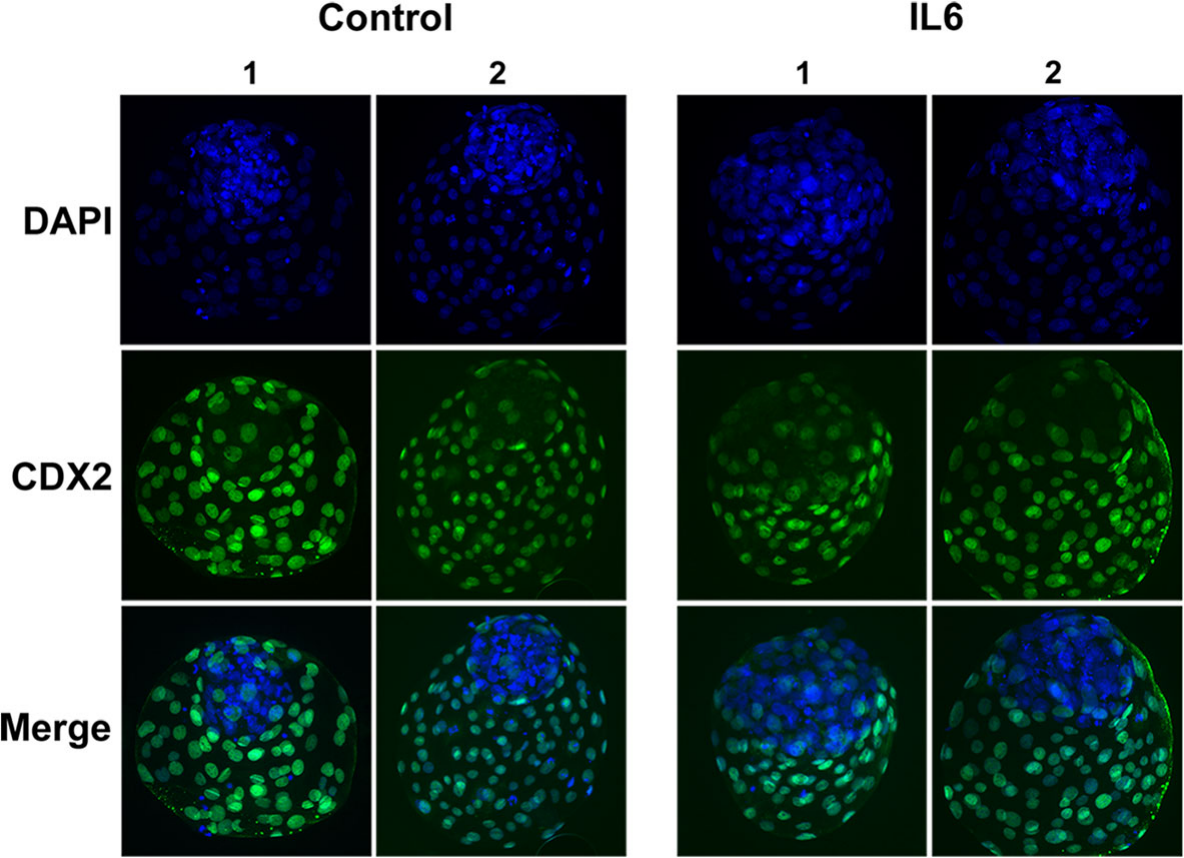
Representative images of differential cell staining in blastocysts collected at day 8 post-fertilization. Embryos either received 0 or 100 ng/ml IL6 beginning at day 5 post-fertilization. *Panel A:* Blastocysts were harvested at day 8, fixed, immunostained, and physically flattened between a slide and coverslip. Photographs represent a single plane of focus. Nuclei representing TE are indicated by CDX2^+^/DAPI^+^ staining (green) and the ICM nuclei are CDX2^−^/DAPI^+^ (blue). Control embryo number 1 had 42 ICM cells and 94 TE cells, while control embryo number 2 had 53 ICM cells and 120 TE cells. IL6-treated embryo number 1 had 86 ICM cells and 99 TE cells, while IL6-treated embryo number 2 had 76 ICM cells and 143 TE cells

Figure 2 provides images of representative blastocysts from the 0 and 100 ng/ml IL6 treatment groups.

### Study B: IL6 treatment at day 3 or 5 post-fertilization

This follow-up study examined whether the 100 ng/ml IL6 concentration could influence blastocyst formation and/or ICM and TE cell numbers when provided at day 3, as the embryonic genome is being activated (8 to 16-cell stages in cattle) [27], and to determine whether this response at day 3 is comparable to providing IL6 at day 5. Also, IL6 administration at both day 3 and 5 (100 ng/ml from day 3 to 5, 200 ng/ml from day 5 to 8) was tested to determine whether this supplementation scheme further improved any outcomes.

Supplementation with 100 ng/ml IL6 either at day 3 or 5 did not affect blastocyst formation at day 7 or 8, but the combined, day 3 and 5 IL6 treatment tended to increase (*P* = 0.06) blastocyst development at day 8 but not day 7 (Table 1). Supplementing IL6 in a single dose at either day 3 or 5 increased ICM cell numbers (*P* < 0.05; Table 2). The combined day 3 and 5 IL6 treatment did not further improve ICM cell numbers. TE cell numbers were not affected by any IL6 treatment. There was a tendency (*P* = 0.09) for IL6 treatment at day 3 to improve total blastomere numbers, but total numbers were not altered in the other treatment groups. Supplementation with IL6 at day 3 or day 5 increased (*P* < 005) the ICM:TE ratio. This ratio was increased further (*P* < 0.05) by supplementing IL6 at both day 3 and 5.

### Study C: IL6 treatment at day 1 post-fertilization

This study began IL6 supplementation at day 1 post-fertilization (i.e. beginning of embryo culture) to test the limits in the duration of IL6 supplementation required to produce ICM responses at day 8. This study design also permitted assessment of how IL6 supplementation affected cleavage rates. A dose-response study was completed to verify previous findings that 100 ng/ml IL6 was required to achieve greater ICM cell numbers.

Cleavage rates tended to be greater (*P* = 0.09) for embryos treated with 1 ng/ml IL6 when compared with controls, but cleavage rates were unaffected by exposure to 10 or 100 ng/ml IL6 (Table 1). Blastocyst formation was unaffected by treatment with 1, 10 or 100 ng/ml IL6. The 100 ng/ml IL6 treatment increased (*P* < 0.05) ICM and total cell numbers in day 8 blastocysts (Table 2). Neither 1 nor 10 ng/ml IL6 affected ICM or total cell numbers. No changes in TE cell numbers were observed with any of the IL6 treatments. Oddly, the ICM:TE ratio differed between the 1 and 10 ng/ml IL6 treatments, but both treatments as well as the controls remained lower than the 100 ng/ml IL6 treatment group (*P* < 0.05).

### Studies D and E: The efficacy of combined IL6 treatments at day 1 and 5 post-fertilization

A subsequent study (Study D) was designed to evaluate whether ICM responses to IL6 could be enhanced further by increasing the dose of IL6 at day 1 (200 ng/ml) or by sequential IL6 supplementation at both days 1 and 5. Media was not exchanged in this treatment group, so these embryos were exposed to 100 ng/ml beginning at day 1 and 200 ng/ml from day 5 to 8. Cleavage and blastocyst rates were not affected by the various IL6 supplementation schemes (Table 1). When compared with the control, ICM and total cell numbers were increased (*P* < 0.05) by supplementing 100 ng/ml IL6 at day 1 or 5 or 200 ng/ml IL6 at day 1 (Table 2). When IL6 was administered on both days 1 and 5, ICM cell numbers but not total cell numbers were greater (*P* < 0.05) than when embryos received IL6 only on day 1 or 5. The number of TE cells were not affected by any treatment. The ICM:TE ratios followed the same response as the ICM cell numbers, where adding 100 ng/ml IL6 at either day 1 or 5 increased (*P* < 0.05) the ICM:TE ratio when compared with the control. Providing 100 ng/ml IL6 at both day 1 and 5 further increased (*P* < 0.05) the ICM:TE ratio. Supplementation with 200 ng/ml IL6 at day 1 provided similar results to when 100 ng/ml IL6 was provided only once during embryo culture. The 200 ng/ml IL6 treatment beginning at day 1 did not produce the same responses as the dual day 1 and 5 IL6 treatment strategy.

Study E was designed to confirm whether the sequential addition of IL6 at day 1 and 5 could be replicated, and to test whether exposure to 200 ng/ml IL6 at day 5 but not at earlier times could produce the same beneficial treatment effect as the dual IL6 treatment strategy. Neither cleavage nor blastocyst development were influenced by the IL6 supplementation schemes examined (Table 1). As before, supplementing 100 ng/ml IL6 at day 5 increased (*P* < 0.05) ICM and total cell numbers when compared with the control (Table 2). However, no further increases in ICM or total cell numbers were detected when the embryos were supplemented with 200 ng/ml IL6 at day 5 or 100 ng/ml IL6 on both days 1 and 5. Again, TE cell numbers were unaffected by IL6 exposure. The ICM:TE ratio was greater (*P* < 0.05) in all IL6-treatments than the control but did not differ from one another.

### Composite analysis of IL6 effects on ICM cell numbers and the ICM:TE ratio

**Fig. 3:**
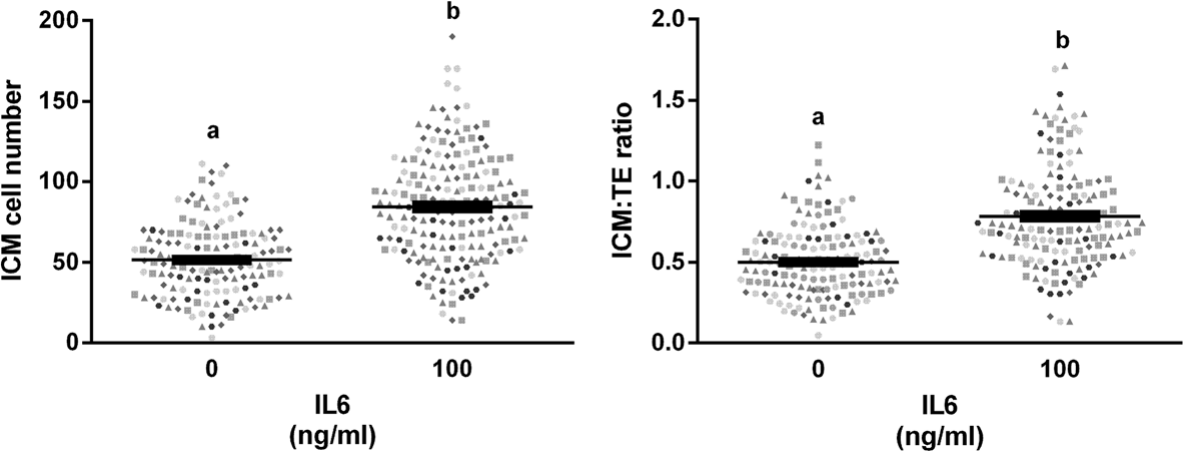
Pooled ICM cell counts and ICM to TE ratios from all studies. All 100 ng/ml treatments of IL6 and associated controls were utilized, regardless of time point of treatment. No other doses of IL6 (1, 10 or 200 ng/ml) are included in this figure. Data from different studies are indicated by different symbols. *Panel A*: Individual ICM counts for embryos receiving either no treatment or 100 ng/ml IL6. *Panel B*: Individual ICM:TE ratios for embryos receiving either no treatment or 100 ng/ml IL6. Corresponding means and SEMs are indicated by the bars. Different superscripts within each panel indicates differences (*P* < 0.05)

A final examination of the effects of 100 ng/ml IL6 supplementation on ICM cell numbers and ICM:TE ratio were explored by graphing each data point from each experiment, regardless of the time when IL6 supplementation was initiated (Fig. 3). An increase in ICM cell numbers and ICM:TE ratio was detected when all data were combined (*P* < 0.0001).

### Study F: IL6 supplementation during individual embryo culture

A final study examined whether IL6 supplementation could overcome the developmental block that occurs when bovine embryos are cultured individually in relatively large drops of culture medium. The IL6 dosages chosen for this study were selected based on a pilot study (data not shown). Individually-cultured zygotes that lacked IL6 supplementation underwent cleavage, albeit at a reduced level (*P* < 0.05) when compared with group-cultured control zygotes (Table 1). None of these individually-cultured control embryos reached the blastocyst stage. By contrast, cleavage rate was not different between group-cultured controls and individually-cultured zygotes supplemented with 100 ng/ml IL4 at both day 1 and 4 or with zygotes supplemented with 200 ng/ml IL6 at day 1. Blastocysts were detected at day 8 but not day 7 in IL6-supplemented, individually-cultured embryos, although the percentage of blastocysts were less than the group-cultured controls (*P* < 0.05). The low number of blastocysts recovered from this study did not permit us to examine how IL6 supplementation affected ICM, TE and total cell numbers and the ICM:TE ratio.

## Discussion

Bovine IVP embryos typically are of lower competency after transfer than in vivo-produced embryos. This is attributed in part to in vitro culture conditions lacking critical embryokines and selective nutrients that the oviduct and uterus produce in early pregnancy. This makes IVP embryos a nice model for investigating ways to improve embryo development and competency in cattle and potentially other species. Work in the mouse and pig has indicated a potential role for IL6 in ICM maintenance [23, 24]. Also, a recent report found that *IL6* transcripts were among the most prominently expressed embryokines in the bovine oviduct and endometrium at day 3 and 5 post-estrus [28–30]. This previous work provided the impetus for us to explore IL6 as an embryokine.

The bovine embryo also produces *IL6* transcripts in both the ICM and TE in blastocysts [25, 31–34]. Our transcript profiling work confirmed the presence of *IL6* transcripts in bovine embryos between the 1-cell and blastocyst stages. We also confirmed the presence of transcripts for both IL6 receptor subunits (*IL6R* and *IL6ST*) throughout early embryo development. *IL6R* was expressed constitutively and was greater in abundance at the 8-cell stage than other stages (excluding the zygote stage). This suggests that *IL6R* transcription ensues as embryonic genome activation begins. No apparent changes in *IL6ST* transcript abundance were detected across the stages examined. However, *IL6ST* mRNA could not be detected in a few of the 2-cell and 8–16 cell embryo samples. We did not pursue if *IL6ST* was truly absent in these samples or if this outcome was caused by using too little RNA. The absence of transcripts also does not guarantee the absence of the mature protein, especially when transcripts were detected at earlier stages of development.

Supplementation with IL6 had no definitive effects on cleavage rates and blastocyst formation when embryos were cultured in groups. This finding contradicts a report in pigs, where improvements in blastocyst development were observed [23]. However, IL6 supplementation was beneficial to embryo development when provided to individually-cultured embryos. A low-density culture environment was employed (1 embryo/ 5 μl medium). This culture scheme usually prevents normal embryo development, presumably because of the lack of conditioning factors that embryos produce in group culture. These positive effects on cleavage and blastocyst rates implicates IL6 as a potential embryokine for mediating embryo development in stressful environments but not when culture conditions are adequate for normal development.

The most notable outcome of this work was observing changes in the composition of blastocysts exposed to IL6 during in vitro embryo development. Improvements in ICM cell numbers were consistently observed after IL6 supplementation, and IL6 promoted ICM development regardless of when it was first administered. In most studies, blastomere numbers within the ICM were nearly doubled in embryos receiving 100 or 200 ng/ml IL6 but not lower IL6 concentrations. Sequential IL6 administration at days 1 and 5 further increased ICM cell numbers in one study (Study D) but failed to do so in another (Study E). This contrast in outcomes may be due solely to chance, although we cannot discount that some unknown factor, such as the genetics of the embryos (ovaries were of unknown origin in every study), may have produced these different responses to sequential IL6 treatment. Regardless, in both studies, this double-treatment scheme still increased ICM cell numbers when compared to the controls.

Individual embryo responsiveness to IL6 varied. However, overwhelmingly positive increases in ICM cell numbers were observed in every study (see Fig. 3). This positive effect of IL6 on ICM cell numbers also was detected in porcine embryos (1.7-fold increase versus controls), suggesting that this phenomenon is not restricted solely to cattle [23].

Another interesting finding from this work was the lack of IL6 effect on TE cell numbers. This explains why substantial increases in the ICM:TE ratio were seen. It also implicates the improvements in ICM cell numbers as the sole reason for the improvements in total embryo cell numbers. The mechanism(s) of action for IL6 are only beginning to be explored, but this work indicates that IL6 solely targets the ICM during early embryogenesis. This observation is consistent with other work that implicates IL6 as a pluripotency factor for mouse embryonic stem cells and for its role in controlling STAT3 activity in early stage mouse embryos [15, 24]. STAT3 is a primary mediator of ICM lineage maintenance in mice [24, 26].

It was also interesting that supplementation with IL6 promoted ICM development regardless of when it was first administered during embryo culture. It was surprising to observe a beneficial effect of IL6 supplementation at day 1. Sufficient amounts of biologically active IL6 may have survived from day 1 to later dates in culture when IL6 could influence embryonic gene expression. The functional lifespan of IL6 was not examined. Alternatively, IL6 provided at day 1 could function post-transcriptionally. More work is needed to clarify this activity.

To enable ICM and TE cell counting, we utilized an immunofluorescence protocol to mark the CDX2-positive TE cells. This is a TE specific marker in bovine blastocysts [35–40]. Binding specificity of the antibody used herein has been verified recently by the absence of staining in CDX2 knock-down bovine blastocysts [41]. We did not utilize an ICM-specific marker. An ICM-specific marker exists for bovine blastocysts, SOX2, however, we chose not to utilize it as this staining is not exclusively nuclear and makes the individual nuclei obscure [42]. Instead, we assumed that CDX2-negative, DAPI-positive nuclei were ICM cells.

We realize the number of blastocysts utilized for cell counting in some treatments and studies appears low (e.g. *n* = 11–16 in study B). In these studies, the effect of 100 or 200 ng/ml IL6 on ICM cell numbers and the ICM:TE ratio is so profound that we do not need to utilize many embryos to detect a difference. Moreover, one can see that this effect can be consistently produced in Fig. 3 (different symbols indicate different studies).

## Conclusions

This work provides evidence that IL6 functions as an embryokine in bovine preimplantation embryos. The beneficial effects of IL6 include increasing ICM blastomere numbers and supporting embryonic development in individual embryo culture systems. The implications of enhancing ICM development in IVP embryos has yet to be explored, but all indications are that IL6 may improve IVP bovine embryo competency, since small ICMs in IVP bovine embryos likely contribute to many of the early pregnancy failures observed in cattle receiving these embryos [6, 10–12].

## Methods

No animals were used for this work. All studies were completed on slaughterhouse-derived materials. Unless specified otherwise, reagents were purchased from ThermoFisher Chemical Company (Waltham, MA).

### In vitro embryo production

Bovine embryos were produced by in vitro maturation, fertilization and culture procedures described previously with some modifications [43, 44]. Cumulus-oocyte complexes (COCs) were harvested from ovaries purchased from Brown Packing Company (Gaffney, SC, USA) or COCs were purchased from DeSoto Biosciences (Seymour, TN) and incubated overnight for 21 to 24 h at 38.5 °C in 5% CO_2_ in groups of 20–35 in 500 μl TCM-199 containing Earle’s salts and supplemented with 10% [*v*/v] fetal bovine serum (FBS; Atlanta Biologicals, Flowery Branch, Georgia, USA), 25 μg/ml bovine follicle stimulating hormone (Bioniche Animal Health Canada Inc., Belleville, Ontario, Canada), 2 μg/ml estradiol (Sigma-Aldrich; St. Louis, MO), 22 μg/ml sodium pyruvate, 1 mM L-alanyl-L-glutamine (Glutamax) and 25 μg/ml gentamicin sulfate. No differences in embryo responses to treatments were observed between COCs harvested in these two manners. For fertilization, COCs were washed in HEPES-SOF and placed in groups of 150–200 in 3 ml SOF-FERT covered by paraffin oil (Ovoil; Vitrolife, Göteborg, Sweden) [43, 45, 46]. Frozen semen from four Holstein bulls (donation from Select Sires, Plain City, OH, USA) was thawed, and spermatozoa were isolated through a biphasic (40 and 80%, [v/v]) Bovipure™ density gradient (Nidacon; Spectrum Technologies Healdsburg, CA, USA) before addition to the fertilization media at a concentration of 1 million sperm/ml fertilization media. Day of fertilization was designated as day 0. After incubation for 14 to 18 h at 38.5 °C in 5% CO_2_ in humidified air, presumptive zygote-cumulus complexes were denuded, washed in HEPES-SOF and, unless otherwise stated, placed in groups of 20–30 in droplets of 50 μl of SOF-BE1 covered by paraffin oil and incubated at 38.5 °C in 5% CO_2_, 5% O_2_ and 90% N_2_ in humidified air [47].

In one study (Study B), embryos visually appraised as healthy (little to no evidence of blastomere degeneration) were harvested at day 3 post-fertilization and transferred to medium containing treatments. For all other studies involving treatments after day 1 post-fertilization, a new treatment method was developed to reduce the amount of handling each embryo experienced and ultimately reduce culture stresses and improve overall development. For this new method, treatments were administered directly to existing drops of embryos via the addition of 2 μl of treatment-concentrated SOF-BE1. This method did not affect treatment outcomes. In studies that began at day 1, presumptive zygotes were transferred directly to medium containing treatment.

### IL6 supplementation studies

For all studies, a concentrated IL6 stock (10 μg/ml, recombinant bovine, Kingfisher Biotech, St. Paul, MN, USA) was prepared in SOF containing 1% [*w*/*v*] bovine serum albumin (BSA; Sigma-Aldrich) and stored in aliquots at − 80 °C. Control treatments consisted of carrier only (1% BSA). Stocks were only thawed once and were used immediately after thawing. Cleavage was assessed at day 3 post-fertilization. Blastocyst formation was recorded at day 7 and 8 post-fertilization.

In the first study (Study A), treatments of 0, 1, 10 or 100 ng/ml IL6 were administered at day 5 post-fertilization (*n* = 20–28 embryos/50 μl drop; 3–4 drops/treatment; 4 replicates). Representative day 8 blastocysts were collected from each treatment group (0, 1, 10 and 100 ng/ml IL6) and processed for cell counting (*n* = 34–39 blastocysts/treatment over 4 replicates).

In the second study (Study B), day 3 embryos (*n* = 80 embryos/treatment; 10 embryos/drop; 6 replicates) were selected and moved to new drops containing either the control treatment or 100 ng/ml IL6. At day 5, half of the drops from each treatment group received 100 ng/ml IL6 via a treatment-concentrated injection to the drops, while the other half received carrier. This created four total treatments: no IL6 (carrier-only), 100 ng/ml IL6 beginning at day 3, 100 ng/ml IL6 beginning at day 5 and 200 ng/ml IL6 administered in 100 ng/ml doses at days 3 and 5. Representative day 8 blastocysts were collected from each treatment group (*n* = 11–16 blastocysts/treatment over 3 replicates) and processed for cell counting.

In the third study (Study C), day 1 embryos were placed in medium containing 0, 1, 10 or 100 ng/ml IL6 (23–26 embryos/drop; 2–4 drops/treatment; 4 replicates). At day 8, representative blastocysts were collected from each treatment group (*n* = 27–41 blastocysts/treatment over 4 replicates) and processed for cell counting.

In the fourth study (Study D), 100 ng/ml IL6 was administered at day 1, day 5, or both day 1 and 5 (total 200 ng/ml IL6), or 200 ng/ml IL6 was administered at day 1. Controls received carrier only at each time point (19–27 embryos/drop; 2–4 drops/treatment/replicate; 6 replicates). At day 8 post-fertilization, representative blastocysts from each treatment group were collected (n = 27–28 blastocysts/treatment over 5 replicates) and processed for cell counting.

In the fifth study (Study E), 0, 100 or 200 ng/ml IL6 was administered at day 5 post-fertilization, or 100 ng/ml was administered on both days 1 and 5 (for a total of 200 ng/ml IL6). Controls received carrier only at both time points (19–30 embryos/drop; 2–6 drops/treatment/replicate; 4 replicates). At day 8 post-fertilization, representative blastocysts from each treatment group were collected (*n* = 38–44 blastocysts/treatment over 4 replicates) and processed for cell counting.

In the sixth study (Study F), embryos were cultured individually (1 embryo/5 μl drop; 25 embryos/treatment; 4 replicates) in medium containing 0, 100 or 200 ng/ml IL6 beginning at day 1. At day 4, each individual culture drop received an additional 1 μl of SOF-BE1 containing either carrier only (for 0 and 200 ng/ml groups) or concentrated IL6 to deliver an additional 100 ng/ml IL6 (for a total of 200 ng/ml IL6 over day 1 and 4). A group-culture control treatment (25 embryos/50 μl drop; 1–2 drops/replicate) lacking IL6 supplementation was also included. Development was assessed at day 4, 7 and 8. No cell counting was completed.

### Transcript profiling

Random samplings of zygotes, 2-cell embryos, 8–16 cell embryos, morulae and blastocysts from the control group (0 ng/ml IL6) were collected at day 1, 2, 4, 6 and 8 post-fertilization, respectively. At each stage, 10 embryos were pooled before RNA extraction. Between 3 to 5 pools of embryos were collected at each stage of development. After washing in Dulbecco’s phosphate-buffered saline (DPBS) containing 0.2% [*w*/*v*] polyvinylpyrrolidone (PBS-PVP), embryos were collected into < 10 μl PBS-PVP in microcentrifuge tubes, snap frozen in liquid nitrogen, and stored at − 80 °C. Total RNA was extracted by using the PicoPure RNA Isolation Kit (Applied Biosystems, Inc., Foster City, CA, USA). The entire RNA sample was incubated with RNase-free DNAse I (20 μl reaction volume) for 30 min at 37 °C followed by 10 min at 75 °C. The RNA (15 μl) was then reverse transcribed using the High Capacity cDNA Reverse Transcription Kit (Applied Biosystems, Inc.) in a total reaction volume of 30 μl. Negative control samples did not receive reverse transcriptase. For PCR, SybrGreen PCR Master Mix (Applied Biosystems, Inc.) was mixed with RT product and 500 nM concentration of forward and reverse primers for a total reaction volume of 10 μl. After activation/denaturation (95 °C for 10 min), a two-step amplification sequence was set for 50 cycles (95 °C for 15 s, 57 °C for 1 min) on an Eppendorf RealPlex 4 MasterCycler. Each sample and primer combination were run in triplicate.

Each primer pair (Table 3) was identified using the Primer-BLAST Program from the National Center for Biotechnology Information (U.S. National Library of Medicine, Bethesda, MD) and synthesized by Integrated DNA Technologies (IDT; San Diego, CA). Primer efficiency standard curve analysis was completed to verify adequate primer efficiency (76–103% efficiency). Dissociation curve analysis (57 to 95 °C) was completed after each PCR amplification to confirm the presence of one amplicon. Succinate dehydrogenase flavoprotein subunit (SDHA) was used as a housekeeping gene based on previously verified stability across early embryonic stages [48]. The abundance of *SDHA* was not influenced by embryo stage in this work. The relative abundance of each target transcript was expressed as fold change from the embryo stage containing the lowest abundance for the specified transcript by using the 2^[-ddCt]^ approach.

**Table 3:** Primers used for quantitative RT-PCR

Gene	Reference or Genbank accession no.	Primer sequence (5′ - 3′)	Product size (bp)
*IL6*	NM_173923.2	Forward: CCAGCCACAAACACTGACCT	121
		Reverse: TAGCTCTCAGGCTGAACTGC	
*IL6R*	NM_001110785.3	Forward: AAGTGCACACCCGTCGTATT	114
		Reverse: TCAGATTCAAGGCTGCTGGG	
*IL6ST*	XM_010816769.3	Forward: GTCTCATGCTCACGGCACTA	220
		Reverse: CGCGTCTGATTTGCCAACAA	
*SDHA*	(Goossens et al. 2005)	Forward: GCAGAACCTGATGCTTTGTG	185
		Reverse: CGTAGGAGAGCGTGTGCTT	

### Immunofluorescence and cell counting

At day 8 post-fertilization, ICM and TE cell numbers were determined in a subset of blastocysts [35]. All blastocysts were utilized when blastocysts numbers were low (< 10/treatment group/replicate). In replicates with large blastocyst numbers, up to 15 blastocysts per treatment were sampled. The selected blastocysts were representative of the types (non-expanded, expanded or hatched) of blastocysts present in each treatment group. On rare occasions (~ 3% of total blastocysts sampled), abnormal blastocysts were identified after staining (< 60 total cells). These blastocysts were excluded from analysis and were considered outliers because their cell numbers were abnormally lower than other blastocysts in this work, and because they were abnormal lower than the expected cell numbers for bovine blastocysts, which should begin blastulation after the 64-cell stage [25]. The incidence of these blastocysts was not restricted to or affected by any one treatment. There were 3 excluded blastocysts in Study A, 5 in Study B, 9 in Study C, 3 in Study D, and 2 in Study E. When assessed by treatment, 3 blastocysts were excluded in the 0 ng/ml treatment, 5 in the 1 ng/ml treatment, 9 in the 10 ng/ml treatment, 3 in the 100 ng/ml treatment, and 2 in the 200 ng/ml treatment group. The numbers of blastocysts examined in Table 2 do not include these abnormal blastocysts.

Embryos were fixed in 4% [*w*/*v*] paraformaldehyde for 15 min at room temperature, permeabilized using 0.25% [*v*/v] Triton-X for 20 min and blocked with 10% [v/v] Horse Serum for 1 h at room temperature. Embryos were then incubated overnight at 4 °C with anti-CDX2 primary antibody (Biogenex, AM392-5 M, sold ready-to-use), washed, and incubated for 1 h at room temperature with either donkey anti-mouse FITC or Alexa Fluor 647 (Invitrogen, A16018 or A31571, 1:200 dilution for either). Embryo DNA was then stained with DAPI (1 μg/ml) for 5 min at room temperature. Embryos were then placed in 10% [v/v] ProLong™ Gold Antifade diluted with PBS-PVP and imaged by flattening on a glass slide lined with a thin layer of petroleum jelly. Immunoreactive complexes and DNA staining were visualized by using an Eclipse Ti-E inverted microscope equipped with an X-Cite 120 epifluorescence illumination system. Images were captured with a DS-L3 digital camera and assembled with NIS-Elements Software (Nikon Instruments, Melville, NY). The program, FIJI (ImageJ) was used to label and record individual nuclei by utilizing the cell counter plugin to count nuclei staining for CDX2 (CDX2^+^, indicating TE) and only DAPI (CDX2^−^/DAPI^+^, indicating ICM) [49].

### Statistical analyses

All analyses except for the individual culture study were completed by least-squares ANOVA using the general linear model of the Statistical Analysis System (Proc GLM; SAS for Windows, version 9.4; SAS Institute Inc., Cary, NC, USA). Relative mRNA abundance data were log-transformed before analysis. For each embryo development study, the IVP replicate was used as the experimental unit. Replicate was considered a random independent variable for all cleavage and blastocyst formation analyses. Percentage data (e.g. blastocyst formation rates) were arcsine-transformed before analysis but are presented as non-transformed means and SEM. The Tukey honestly significant difference test was used for all cleavage and blastocyst formation data. Blastomere numbers and differential staining analysis used individual embryos as the experimental unit. Individual comparisons of blastomere numbers were partitioned further by using the Probability of Difference (PDIFF) test of SAS. Chi-square analysis was used to analyze the individual embryo culture study. Statistical significance was determined at *P* ≤ 0.05.

## Abbreviations

COCs: Cumulus-oocyte complexes
ICM: Inner cell mass
IL6: Interleukin-6
IL6R: Interleukin-6 receptor
IL6ST: Interleukin-6 signal transducer (aka gp130)
IVP: In-vitro produced
LIF: Leukemia inhibitory factor
SDHA: Succinate dehydrogenase flavoprotein subunit
STAT3: Signal transducer and activator of transcription 3
TE: Trophectoderm

## References

[1] Feldman B, Poueymirou W, Papaioannou V, DeChiara T, Goldfarb M (1995). Requirement of FGF-4 for postimplantation mouse development. Science.

[2] Arman E, Haffner-Krausz R, Chen Y, Heath JK, Lonai P (1998). Targeted disruption of fibroblast growth factor (FGF) receptor 2 suggests a role for FGF signaling in pregastrulation mammalian development. Proc Natl Acad Sci.

[3] den Abbeel E, Balaban B, Ziebe S, Lundin K, Cuesta M, Klein B (2013). Association between blastocyst morphology and outcome of single-blastocyst transfer. Reprod BioMed Online.

[4] Kovacic B, Vlaisavljevic V, Reljic M, Cizek-Sajko M (2004). Developmental capacity of different morphological types of day 5 human morulae and blastocysts. Reprod BioMed Online.

[5] Richter KS, Harris DC, Daneshmand ST, Shapiro BS (2001). Quantitative grading of a human blastocyst: optimal inner cell mass size and shape. Fertil Steril.

[6] Fischer-Brown A, Lindsey B, Ireland F, Northey D, Monson R, Clark S (2004). Embryonic disc development and subsequent viability of cattle embryos following culture in two media under two oxygen concentrations. Reprod Fertil Dev.

[7] Peterson A, Lee R-F (2003). Improving successful pregnancies after embryo transfer. Theriogenology.

[8] Iwasaki S, Yoshiba N, Ushijima H, Watanabe S, Nakahara T (1990). Morphology and proportion of inner cell mass of bovine blastocysts fertilized in vitro and in vivo. J Reprod Fertil.

[9] Bertolini M, Beam SW, Shim H, Bertolini LR, Moyer AL, Famula TR (2002). Growth, development, and gene expression by in vivo- and in vitro-produced day 7 and 16 bovine embryos. Mol Reprod Dev.

[10] Loureiro B, Block J, Favoreto MG, Carambula S, Pennington KA, Ealy AD (2011). Consequences of conceptus exposure to colony-stimulating factor 2 on survival, elongation, interferon-τ secretion, and gene expression. Reproduction (Cambridge, England).

[11] Machado GM, Ferreira AR, Pivato I, Fidelis A, Spricigo JF, Paulini F (2013). Post-hatching development of in vitro bovine embryos from day 7 to 14 in vivo versus in vitro. Mol Reprod Dev.

[12] Alexopoulos NI, French AJ (2009). The prevalence of embryonic remnants following the recovery of post-hatching bovine embryos produced in vitro or by somatic cell nuclear transfer. Anim Reprod Sci.

[13] Scheller J, Chalaris A, Schmidt-Arras D, Rose-John S (2011). The pro- and anti-inflammatory properties of the cytokine interleukin-6. Biochimica et Biophysica Acta (BBA) - Molecular Cell Research.

[14] Kimber SJ (2005). Leukaemia inhibitory factor in implantation and uterine biology. Reproduction.

[15] Yoshida K, Chambers I, Nichols J, Smith A, Saito M, Yasukawa K (1994). Maintenance of the pluripotential phenotype of embryonic stem cells through direct activation of gp130 signalling pathways. Mech Dev.

[16] Williams R, Hilton D, Pease S, Willson T, Stewart C, Gearing D (1988). Myeloid leukaemia inhibitory factor maintains the developmental potential of embryonic stem cells. Nature.

[17] Nichols J, Chambers I, Smith A (1994). Derivation of germline competent embryonic stem cells with a combination of Interleukin-6 and soluble Interleukin-6 receptor. Exp Cell Res.

[18] Heinrich PC, Behrmann I, MÜller-Newen G, Schaper F, Graeve L (1998). Interleukin-6-type cytokine signalling through the gp130/Jak/STAT pathway. Biochem J.

[19] Kocyigit A, Cevik M (2015). Effects of leukemia inhibitory factor and insulin-like growth factor-I on the cell allocation and cryotolerance of bovine blastocysts. Cryobiology.

[20] Sirisathien S, Hernandez-Fonseca H, Bosch P, Hollet B, Lott J, Brackett B (2003). Effect of leukemia inhibitory factor on bovine embryos produced in vitro under chemically defined conditions. Theriogenology.

[21] Rodriguez A, Frutos DC, Diez C, Caamaño J (2007). Effects of human versus mouse leukemia inhibitory factor on the in vitro development of bovine embryos.

[22] Vejlsted M, Avery B, Gjorret J, Maddox-Hyttel P (2005). Effect of leukemia inhibitory factor (LIF) on in vitro produced bovine embryos and their outgrowth colonies. Mol Reprod Dev.

[23] Shen X, Cui X, Lee S, Kim N (2012). Interleukin-6 enhances porcine parthenote development in vitro, through the IL-6/Stat3 signaling pathway. J Reprod Dev.

[24] Do D, Ueda J, Messerschmidt D, Lorthongpanich C, Zhou Y, Feng B (2013). A genetic and developmental pathway from STAT3 to the OCT4-NANOG circuit is essential for maintenance of ICM lineages in vivo. Genes Dev.

[25] Hosseini DI, Caballero J, Moulavi F, Ghanaei H, Sirard M (2015). Transcriptome profiling of bovine inner cell mass and trophectoderm derived from in vivo generated blastocysts. BMC Dev Biol.

[26] Takeda K, Noguchi K, Shi W, Tanaka T, Matsumoto M, Yoshida N (1997). Targeted disruption of the mouse Stat3 gene leads to early embryonic lethality. Proc Natl Acad Sci U S A.

[27] Graf A, Krebs S, Heininen-Brown M, Zakhartchenko V, Blum H, Wolf E (2014). Genome activation in bovine embryos: review of the literature and new insights from RNA sequencing experiments. Anim Reprod Sci.

[28] Fischer C, Drillich M, Odau S, Heuwieser W, Einspanier R, Gabler C (2010). Selected pro-inflammatory factor transcripts in bovine endometrial epithelial cells are regulated during the oestrous cycle and elevated in case of subclinical or clinical endometritis. Reprod Fertil Dev.

[29] Healy LL, Cronin JG, Sheldon IM. Polarized Epithelial Cells Secrete Interleukin 6 Apically in the Bovine Endometrium. Biol Reprod. 2015;92(6):151. 10.1095/biolreprod.115.127936. 10.1095/biolreprod.115.127936 25740541

[30] Tríbulo P, Siqueira LGB, Oliveira LJ, Scheffler T, Hansen PJ (2018). Identification of potential embryokines in the bovine reproductive tract. J Dairy Sci.

[31] Mamo S, Mehta J, Forde N, McGettigan P, Lonergan P. Conceptus-endometrium crosstalk during maternal recognition of pregnancy in cattle. Biol Reprod. 2012;87(6):1–9. 10.1095/biolreprod.112.099945 22517619

[32] Jiang Z, Sun J, Dong H, Luo O, Zheng X, Obergfell C, et al. Transcriptional profiles of bovine in vivo pre-implantation development. BMC Genomics. 2014;15:756. 10.1186/1471-2164-15-756 PMC4162962 25185836

[33] Graf A, Krebs S, Zakhartchenko V, Schwalb B, Blum H, Wolf E. Fine mapping of genome activation in bovine embryos by RNA sequencing. Proc Natl Acad Sci U S A. 2014;111:4139–44. 10.1073/pnas.1321569111 PMC3964062 24591639

[34] Mathialagan N, Bixby J, Roberts R (1992). Expression of interleukin-6 in porcine, ovine, and bovine preimplantation conceptuses. Mol Reprod Dev.

[35] Yang Q-E, Ozawa M, Zhang K, Johnson SE, Ealy AD (2014). The requirement for protein kinase C delta (PRKCD) during preimplantation bovine embryo development. Reprod Fertil Dev.

[36] Kuijk EW, Puy L, Tol H, Oei C, Haagsman HP, Colenbrander B (2008). Differences in early lineage segregation between mammals. Dev Dyn.

[37] Negrón-Pérez VM, Hansen PJ. Role of yes-associated protein 1, Angiomotin and mitogen activated kinase kinase 1/2 in development of the bovine blastocyst. Biol Reprod. 2017;98(2):170-183. 10.1093/biolre/iox172. 10.1093/biolre/iox172 PMC6669417 29228123

[38] Negrón-Pérez VM, Vargas-Franco D, Hansen PJ (2017). Role of chemokine (C-C motif) ligand 24 in spatial arrangement of the inner cell mass of the bovine embryo†. Biol Reprod.

[39] Simmet K, Zakhartchenko V, Philippou-Massier J, Blum H, Klymiuk N, Wolf E (2018). OCT4/POU5F1 is required for NANOG expression in bovine blastocysts. Proc Natl Acad Sci.

[40] Valleh M, Hyttel P, Rasmussen M, Strøbech L (2014). Insulin-like growth factor 2: a modulator of anti-apoptosis related genes (HSP70, BCL2-L1) in bovine preimplantation embryos. Theriogenology.

[41] Wu X, Song M, Yang X, Liu X, Liu K, Jiao C (2016). Establishment of bovine embryonic stem cells after knockdown of CDX2. Sci Rep.

[42] Goissis MD, Cibelli JB (2014). Functional characterization of SOX2 in bovine preimplantation embryos. Biol Reprod.

[43] Xie M, McCoski SR, Johnson SE, Rhoads ML, Ealy AD. Combinatorial effects of epidermal growth factor, fibroblast growth factor 2 and insulin-like growth factor 1 on trophoblast cell proliferation and embryogenesis in cattle. Reprod Fertil Dev. 2017;29(2):419-30. 10.1071/RD15226. 10.1071/RD15226 26304178

[44] Zhang K, Hansen PJ, Ealy AD (2010). Fibroblast growth factor 10 enhances bovine oocyte maturation and developmental competence in vitro. Reproduction.

[45] Denicol AC, Block J, Kelley DE, Pohler KG, Dobbs KB, Mortensen CJ (2014). The WNT signaling antagonist Dickkopf-1 directs lineage commitment and promotes survival of the preimplantation embryo. FASEB J.

[46] Sakatani ANV, Takahashi M, Hansen PJ (2012). Consequences of physiological heat shock beginning at the zygote stage on embryonic development and expression of stress response genes in cattle. J Dairy Sci.

[47] Fields SD, Hansen PJ, Ealy AD (2011). Fibroblast growth factor requirements for in vitro development of bovine embryos. Theriogenology.

[48] Goossens K, Poucke M, Soom A, Vandesompele J, Zeveren A, Peelman LJ (2005). Selection of reference genes for quantitative real-time PCR in bovine preimplantation embryos. BMC Dev Biol.

[49] Schindelin J, Arganda-Carreras I, Frise E, Kaynig V, Longair M, Pietzsch T (2012). Fiji: an open-source platform for biological-image analysis. Nat Methods.

